# Distinguishing SARS-CoV-2 persistence and reinfection: A retrospective cohort study

**DOI:** 10.1093/cid/ciac830

**Published:** 2022-10-21

**Authors:** Sarah E Turbett, Christopher H Tomkins-Tinch, Melis N Anahtar, Caitlin M Dugdale, Emily P Hyle, Erica S Shenoy, Bennett Shaw, Kenechukwu Egbuonu, Kathryn A Bowman, Kimon C Zachary, Gordon C Adams, David C Hooper, Edward T Ryan, Regina C LaRocque, Ingrid V Bassett, Virginia A Triant, Katherine J Siddle, Eric Rosenberg, Pardis C Sabeti, Stephen F Schaffner, Bronwyn L MacInnis, Jacob E Lemieux, Richelle C Charles

**Affiliations:** Division of Infectious Diseases, Department of Medicine, Massachusetts General Hospital, Boston, MA, USA; Harvard Medical School, Boston, MA, USA; Department of Pathology, Massachusetts General Hospital, Boston, MA, USA; Broad Institute of MIT and Harvard, Cambridge, MA, USA; Department of Organismic and Evolutionary Biology, Harvard University, Cambridge, MA, USA; Department of Pathology, Massachusetts General Hospital, Boston, MA, USA; Broad Institute of MIT and Harvard, Cambridge, MA, USA; Division of Infectious Diseases, Department of Medicine, Massachusetts General Hospital, Boston, MA, USA; Harvard Medical School, Boston, MA, USA; Medical Practice Evaluation Center, Department of Medicine, Massachusetts General Hospital, Boston, MA, USA; Division of Infectious Diseases, Department of Medicine, Massachusetts General Hospital, Boston, MA, USA; Harvard Medical School, Boston, MA, USA; Medical Practice Evaluation Center, Department of Medicine, Massachusetts General Hospital, Boston, MA, USA; Division of Infectious Diseases, Department of Medicine, Massachusetts General Hospital, Boston, MA, USA; Harvard Medical School, Boston, MA, USA; Infection Control Unit, Massachusetts General Hospital, Boston, MA, USA; Division of Infectious Diseases, Department of Medicine, Massachusetts General Hospital, Boston, MA, USA; Broad Institute of MIT and Harvard, Cambridge, MA, USA; David Geffen School of Medicine at the University of California Los Angeles, Los Angeles, CA, USA; Massachusetts Institute of Technology, Boston, Massachusetts, USA; Division of Infectious Diseases, Department of Medicine, Massachusetts General Hospital, Boston, MA, USA; Harvard Medical School, Boston, MA, USA; Ragon Institute of MGH, MIT, and Harvard, Cambridge, Massachusetts, USA; Division of Infectious Diseases, Department of Medicine, Massachusetts General Hospital, Boston, MA, USA; Harvard Medical School, Boston, MA, USA; Infection Control Unit, Massachusetts General Hospital, Boston, MA, USA; Broad Institute of MIT and Harvard, Cambridge, MA, USA; Division of Infectious Diseases, Department of Medicine, Massachusetts General Hospital, Boston, MA, USA; Harvard Medical School, Boston, MA, USA; Infection Control Unit, Massachusetts General Hospital, Boston, MA, USA; Division of Infectious Diseases, Department of Medicine, Massachusetts General Hospital, Boston, MA, USA; Harvard Medical School, Boston, MA, USA; Department of Immunology and Infectious Diseases, Harvard T.H. Chan School of Public Health, Harvard University, Boston, Massachusetts, USA; Division of Infectious Diseases, Department of Medicine, Massachusetts General Hospital, Boston, MA, USA; Harvard Medical School, Boston, MA, USA; Division of Infectious Diseases, Department of Medicine, Massachusetts General Hospital, Boston, MA, USA; Harvard Medical School, Boston, MA, USA; Medical Practice Evaluation Center, Department of Medicine, Massachusetts General Hospital, Boston, MA, USA; Division of Infectious Diseases, Department of Medicine, Massachusetts General Hospital, Boston, MA, USA; Harvard Medical School, Boston, MA, USA; Division of General Internal Medicine, Department of Medicine, Massachusetts General Hospital, Boston, Massachusetts, USA; Broad Institute of MIT and Harvard, Cambridge, MA, USA; Division of Infectious Diseases, Department of Medicine, Massachusetts General Hospital, Boston, MA, USA; Harvard Medical School, Boston, MA, USA; Department of Pathology, Massachusetts General Hospital, Boston, MA, USA; FAS Center for Systems Biology, Harvard University, Boston, MA, USA; Broad Institute of MIT and Harvard, Cambridge, MA, USA; Department of Organismic and Evolutionary Biology, Harvard University, Cambridge, MA, USA; Department of Immunology and Infectious Diseases, Harvard T.H. Chan School of Public Health, Harvard University, Boston, Massachusetts, USA; Broad Institute of MIT and Harvard, Cambridge, MA, USA; Department of Immunology and Infectious Diseases, Harvard T.H. Chan School of Public Health, Harvard University, Boston, Massachusetts, USA; Division of Infectious Diseases, Department of Medicine, Massachusetts General Hospital, Boston, MA, USA; Harvard Medical School, Boston, MA, USA; Broad Institute of MIT and Harvard, Cambridge, MA, USA; Division of Infectious Diseases, Department of Medicine, Massachusetts General Hospital, Boston, MA, USA; Harvard Medical School, Boston, MA, USA; Department of Immunology and Infectious Diseases, Harvard T.H. Chan School of Public Health, Harvard University, Boston, Massachusetts, USA

**Keywords:** COVID-19, SARS-CoV-2 reinfection, persistent detection

## Abstract

**Background:**

SARS-CoV-2 reinfection is poorly understood, partly because few studies have systematically applied genomic analysis to distinguish reinfection from persistent RNA detection related to initial infection. We aimed to evaluate the characteristics of SARS-CoV-2 reinfection and persistent RNA detection using independent genomic, clinical, and laboratory assessments.

**Methods:**

All individuals at a large academic medical center who underwent a SARS-CoV-2 nucleic acid amplification test (NAAT) ≥ 45 days after an initial positive test, with both tests between March 14^th^ and December 30^th^, 2020, were analyzed for potential reinfection. Inclusion criteria required having ≥2 positive NAATs collected ≥45 days apart with a cycle threshold (Ct) value <35 at repeat testing. For each included subject, likelihood of reinfection was assessed by viral genomic analysis of all available specimens with a Ct value <35, structured Ct trajectory criteria, and case-by-case review by infectious diseases physicians.

**Results:**

Among 1,569 individuals with repeat SARS-CoV-2 testing ≥45 days after an initial positive NAAT, 65 (4%) met cohort inclusion criteria. Viral genomic analysis characterized mutations present, and was successful for 14/65 (22%) subjects. Six subjects had genomically-supported reinfection and eight subjects had genomically-supported persistent RNA detection. Compared to viral genomic analysis, clinical and laboratory assessments correctly distinguished reinfection from persistent RNA detection in 12/14 (86%) subjects but missed 2/6 (33%) genomically-supported reinfections.

**Conclusion:**

Despite good overall concordance with viral genomic analysis, clinical and Ct value-based assessments failed to identify 33% of genomically-supported reinfections. Scaling-up genomic analysis for clinical use would improve detection of SARS-CoV-2 reinfections.

## Teaser

We evaluated characteristics of SARS-CoV-2 reinfection and persistent RNA detection using genomic, clinical, and laboratory assessments. Despite overall good concordance among these three approaches in identifying persistent RNA detection, clinical and laboratory assessments failed to identify 33% of genomically-supported reinfections.

## Introduction

Nucleic acid amplification tests (NAATs) can detect SARS-CoV-2 RNA weeks to months after initial infection [1, 2]. Therefore, positive NAATs obtained several weeks after an initial diagnosis pose a clinical dilemma – these results may reflect delayed clearance of non-viable virus, chronic active infection with viable virus, or SARS-CoV-2 reinfection, each of which carries different implications for treatment, contact tracing, and transmission-based precautions [3-6]. In addition to aiding clinical decision-making, distinguishing reinfection from persistent RNA detection related to the initial infection is of public health importance, because reinfections may indicate viral escape due to waning immunity or signal the emergence of novel variants [5, 7]. However, without viral genomic analysis, which is not yet widely available for clinical use, it is challenging to distinguish SARS-CoV-2 reinfection from persistent RNA detection using routinely available clinical and laboratory data alone [3-5].

Previous attempts to identify SARS-CoV-2 reinfection incidence and associated clinical characteristics have mostly relied on time-based definitions for reinfection (i.e., having a repeat positive test at some interval after diagnosis), rather than genomic analysis, leading to potential misclassification of reinfection cases [2, 8-10]. The Centers for Disease Control and Prevention (CDC) Investigative Criteria for Suspected Cases of SARS-CoV-2 Reinfection prioritizes genomic evaluation of viral specimens from individuals with a repeat positive NAAT ≥45 days after initial diagnosis to distinguish reinfection from persistent RNA detection (Appendix Table 1) [11]. These criteria have been applied to many individual cases [12-14], but they have not been used to systematically identify SARS-CoV-2 reinfection in large, well-characterized cohorts.

We report clinical, laboratory, and genomic characterization of a large cohort of individuals with repeat positive NAATs ≥45 days after initial COVID-19 diagnosis at an academic medical center in Boston. We also describe the strengths and limitations of clinical, laboratory, and genomics-based assessment for the identification of SARS-CoV-2 reinfection in the clinical setting.

**Methods**

*Data sources and inclusion criteria*

We queried the Massachusetts General Hospital (MGH) electronic health record to identify subjects with at least one SARS-CoV-2 NAAT collected at an MGH facility ≥45 days after any prior positive NAAT (Supplementary Appendix Methods). Subjects were included in the study cohort if they had a subsequent positive NAAT collected ≥45 days after a prior positive NAAT that (1) was from a nasopharyngeal (NP) or anterior nares (AN) specimen, (2) had a cycle threshold (Ct) value <35 from any target, and (3) had residual specimen available for genomic analysis. These criteria were adapted from the CDC SARS-CoV-2 reinfection investigation criteria (Appendix Table 1). This study was approved by the Mass General Brigham Institutional Review Board (2019P003305).

*Clinical review*

We obtained demographic and clinical data regarding the initial infection episode among subjects hospitalized at MGH with COVID-19 during the first surge from March 11, 2020 – June 3, 2020 from the MGH COVID-19 Registry [15]. For these subjects' subsequent testing encounters and for all data on subjects who were not hospitalized at MGH with COVID-19

during the first surge, we extracted demographic, clinical, and laboratory data from the electronic health record into a standardized data collection form.

To assess clinical suspicion for COVID-19 reinfection, two infectious disease physician reviewers conducted detailed chart review and categorized each subject into either “low” or “moderate to high” clinical suspicion for reinfection. Reviewers were blinded to subjects’ Ct values, genomic data, and other reviewers’ categorizations. Features that favored “moderate to high” suspicion for reinfection included: new or recurrent upper respiratory infection symptoms, no clear alternative diagnosis, reporting a COVID-19 contact, and/or imaging and laboratory findings consistent with acute COVID-19 infection. All subjects also underwent Biofire™ extended respiratory viral testing (Supplemental Appendix Methods); however, these results were not available to reviewers unless obtained as part of clinical care. Initial clinical assessment categorizations were discordant for 6/65 (9%) subjects and were adjudicated by a third, blinded reviewer.

*Ct value assessments*

For each included subject, we reviewed all available SARS-CoV-2 NAAT results and associated Ct values. Ct values obtained from the TaqPath® COVID-19 Combo Kit were standardized to account for variability across testing platforms (Supplementary Appendix Methods and Appendix Figure 1) [16]. Ct value assessments of reinfection were assigned to one of four categories - strongly supportive, supportive, not supportive, or inconclusive - using pre-defined criteria based on established viral kinetics of SARS-CoV-2 infection (Appendix Table 2) [2]. We compared the yield of genomic data with specimen Ct values <30 vs. ≥30 using a Fisher's Exact test, considering a 2-sided *P*-value <0.05 to be statistically significant.

*Genomic analysis*

We performed either randomly-primed metagenomic sequencing using NexteraXT (41 samples) or tiled-amplicon sequencing with ARTICv3 primers (156 samples) on each thawed sample, sequencing the libraries as members of multiplexed pools (Supplementary Appendix Methods). Samples from most subjects did not produce complete viral genomes at all time points, which limited the ability to use only a change in PANGO lineage designation to determine reinfection. Therefore, support for reinfection was corroborated based on the following criteria, consistent with CDC guidance [11], using initial and subsequent infection period genomes: 1) well-supported phylogenetic separation (i.e., change in PANGO lineage or a Shimodaira-Hasegawa likelihood ratio test node support metric >80%), 2) a higher-than-expected substitution rate for the population average (>2 substitutions/30 days), or 3) presence of a PANGO lineage during the subsequent episode that was not in local circulation (i.e. <1% prevalence) during initial infection (Supplementary Appendix Methods) [11, 13, 17, 18]. Conversely, subjects whose later viral genomes descended from earlier genomes and had substitution rates less than the population average were deemed to have persistent RNA detection (i.e., no reinfection) [11].

*Determining the likelihood of reinfection*

We stratified included subjects into one of five categories. Regardless of clinical or Ct value assessments, subjects with genomic demonstration of reinfection were categorized as *genomically-supported reinfection*. Similarly, subjects with genomic evidence of persistent RNA

detection were classified as *genomically-supported persistent RNA detection* (no reinfection). If genomic analysis was indeterminate, categorization was based on clinical and Ct value assessment criteria into *probable reinfection, probable persistent RNA detection* (unlikely reinfection), or *inconclusive* (Appendix Table 3).

### Results

*Identifying included subjects using time-based criteria*

Between March 14, 2020 and December 30, 2020, 1,569 individuals had at least one SARS-CoV-2 NAAT collected at MGH ≥45 days after a prior positive test (Figure 1A), (Supplementary Appendix Methods). Among individuals with repeat testing ≥45 days after their initial positive test, 195/1569 (12%) had a positive repeat test (Figure 1B). Of these 195 subjects, 130 (67%) were excluded, because their repeat tests had a Ct value that was either ≥35 (120 subjects) or not available (6 subjects), were collected from the lower respiratory tract (3 subjects), or were negative when repeated to assess study eligibility (1 subject). In total, 65/1,569 (4%) subjects with repeat testing were included in the cohort.

*Yield of genomic analysis*

SARS-CoV-2 sequencing was attempted on all available positive NP or AN specimens with a Ct <35 (n=197) from the 65 subjects in the cohort, producing 170 partial or complete viral genome assemblies (Appendix Figure 2). For 14/65 (22%) subjects, the quality of genomic data was sufficiently high to assess reinfection (Figure 1B). The 51 remaining subjects could not be genomically classified because of specimen unavailability (16/51, 31%) or insufficient genome coverage (35/51, 69%). Specimens with Ct values >30 were less likely to yield high quality genomic data than those with Ct $\leq$30 (p-value <0.0001, Appendix Figure 2 and 3), consistent with previous reports [19, 20].

*Genomics-based classification and analysis*

For the 14 subjects with sufficient genomic data at multiple time points, we identified six cases of *genomically-supported reinfection* (Figure 2) and eight cases of *genomically-supported persistent RNA detection* (Figure 3). Of the six *genomically-supported reinfection* cases, three (Subjects R1, R4, and R5) were classified as reinfection with moderate genomic support, as time series genome data was unavailable but the viral PANGO lineage detected in the later time point had not been detected globally (R1 and R5) or was not yet in circulation in Massachusetts (R4) during the initial time period of positivity (Figure 2), making initial infection with the lineages highly improbable. The remaining three cases were classified as reinfections with strong genomic support. Two cases (Subjects R3 and R6) were identified as *genomically-supported reinfections* based on the phylogenetic separation of early and later genomes (i.e. later genomes descended from other cases rather than from the earlier viral genomes); these also exhibited a greater than average nucleotide substitution rate, consistent with reinfection [11]. These two cases exhibited a return to the ancestral allele in later timepoints at several polymorphic positions. The last case (Subject R2) was identified based on phylogenetic separation as well as a subtle lineage change from B.1 to B.1.1. In contrast, the eight subjects categorized as *persistent RNA detection* showed no genomic evidence for reinfection.

Among *genomically-supported reinfection* cases, we identified mutations in the genomes from second infections relative to the ancestral reference genome (Appendix Figure 4, Appendix

Table 4). Several of these mutations were non-synonymous substitutions of potential functional relevance. Subject R1 had the *Mustelidae*-associated substitution S:N501T in the spike protein [21, 22], one also reported in a prior reinfection case [13]. Subject R6 had the substitution S:Q677H near the furin cleavage site of the spike protein [23, 24] a recurrent emergence on several distant lineages [25]. Subject R2 exhibited the substitutions N:R203K and N:G204R, observed to replicate well without cytopathy, subsequently seen in the Alpha, Gamma, and Omicron lineages, and found to increase viral particle production by elevating nucleocapsid expression [26, 27].

We characterized viral genetic changes that occurred during infection in the eight subjects with *genomically-supported persistent RNA detection* (Subjects P1-P8; Figure 3; Appendix Figure 4; Appendix Table 4), to examine viral evolution within the host. Subjects P1, P3, P4, P6, P7, and P8 exhibited viral substitutions in the middle of each time series that were not seen in the first or final time points, consistent with mixed viral populations and the emergence and disappearance of subclones, as previously reported [28]. The average apparent substitution rate of genomes among all subjects with genomically-supported persistent RNA detection (0.9 substitutions per 30 days) was below the average rate reported for the virus across the broader population [17].

*Concordance between genomic findings and clinical and Ct value assessments*

Among the six subjects with *genomically-supported reinfection*, the median age was 34 years (IQR: 28, 45), five (83%) were male, and none were immunocompromised (Table 1). Median Ct values were higher for the first NAAT compared to the subsequent qualifying NAAT (32.7 [IQR: 25.0, 33.6] vs. 26.4 [IQR: 22.7, 30.8]). In four of six (67%) individuals, there was either

“moderate to high" clinical suspicion or strong Ct value trajectory support for reinfection (Appendix Figure 5, Figure 2), with three of six (50%) cases satisfying both criteria. Subjects R1, R4, and R6 had mild, typical COVID-19 symptoms with the second illness episode. Subject R3 had respiratory symptoms, which were attributed to post-obstructive pneumonia, and he subsequently died of lung cancer complications. Subjects R2 and R5 were asymptomatic, but Ct values were strongly suggestive of reinfection for Subject R5. For Subjects R2 and R3, clinical and Ct value assessment alone was insufficient to identify reinfection.

Among the eight subjects with *genomically-supported persistent RNA detection*, the median age was 40 years (IQR: 24, 64), five (63%) were male, and four (50%; Subjects P1, P2, P3, P6) were immunocompromised (Table 1). Median Ct values were lower for the first NAAT (19.2 [IQR: 16.7, 23.7]) compared to the subsequent qualifying NAAT (32.7 [IQR: 29.1, 34.2]). There was low suspicion for reinfection based on clinical and Ct value assessments in all eight subjects (Figure 3). One immunocompromised subject receiving rituximab (Subject P1) had persistently positive NAATs with moderate Ct values (range 25-29) up to 70 days after initial infection [29].

*Clinical and Ct value assessments among subjects not genomically classified*

When subjects could not be genomically classified (51/65 subjects, 78%), we used clinical and Ct value assessments to classify subjects as *probable reinfection, probable persistent RNA detection* (unlikely reinfection), or *inconclusive* (Appendix Tables 3 and 5). Eight (12%) subjects were categorized as *probable reinfection* based on “moderate to high" clinical suspicion with a supportive Ct value assessment, or with a strongly supportive Ct value assessment alone (Appendix Figure 6). Subjects with *probable reinfection* had a median duration of 87 days (IQR:

77, 111) between the first and repeat qualifying positive NAAT and lower median Ct values at repeat compared to initial testing (27.8 [IQR: 21.0, 34.1] versus 32.3 [IQR: 31.2, 32.4]; Table 1, Figure 4). Conversely, we classified 18 subjects (28%) as *probable persistent RNA detection* based on "low" clinical suspicion for reinfection and a Ct value assessment that was not supportive of reinfection (Appendix Figure 7). These subjects had a median of 55 days (IQR: 50, 65) between the first and repeat qualifying positive NAATs, and higher median Ct values at repeat compared to initial testing (33.8 [IQR: 33.5, 34.7] versus 22.9 [IQR: 15.8, 31.2]). The remaining 25 subjects (38%) had inconclusive results and could not be categorized due to insufficient data or discordant clinical and Ct value assessments (Appendix Figure 8).

### Discussion

As SARS-CoV-2 reinfection increases in the era of Omicron and other variants of concern [7, 30], so does the importance of promptly and accurately distinguishing SARS-CoV-2 reinfection from persistent RNA detection after initial infection. Failing to recognize SARS-CoV-2 reinfection may result in missed opportunities for initiation of COVID-19 treatment, onward transmission, and delayed detection of novel SARS-CoV-2 variants [3-5, 7]. In contrast, approaching all individuals with positive NAATs long after initial infection as having reinfection places increased pressure on strained healthcare systems [31, 32] by risking misallocation of therapeutics, private hospital rooms, and personal protective equipment. While the stakes are high, standardized approaches to distinguish reinfection from persistent RNA detection are lacking.

We systematically used clinical, laboratory, and genomic information to distinguish SARS-CoV-2 reinfection from persistent RNA detection among individuals who underwent serial SARS-CoV-2 NAATs as part of routine care. Cases of *genomically-supported reinfection* were often mild or asymptomatic, despite generally low Ct values on repeat testing, suggesting transmissibility [33, 34]. We found that a third (2/6) of individuals with *genomically-supported reinfection* would have been misclassified with clinical and Ct value assessment alone, underscoring the challenge of distinguishing between SARS-CoV-2 reinfection and persistence using routinely available clinical and laboratory data. Conversely, clinical and Ct value assessment accurately identified all (8/8) individuals with *genomically-supported persistent RNA detection* (i.e., no reinfection).

We found that structured clinical and Ct value assessments performed reasonably well in distinguishing reinfection from persistent RNA detection, demonstrating 86% (12/14 subjects) concordance with viral genomic analysis, but these real-time assessments have some limitations. SARS-CoV-2 reinfection is frequently asymptomatic and may be missed by clinical assessment [35, 36]. Low or decreasing Ct values can raise suspicion for reinfection. However, Ct values vary by time from symptom onset, testing platform, and sample collection, transport, and storage conditions [37], complicating Ct value interpretation. Despite these limitations, our findings suggest that clinicians can distinguish reinfection from persistent RNA detection with reasonable accuracy by integrating clinical and Ct value assessments with established time-based definitions of reinfection.

This study demonstrates that viral genomic analysis can improve sensitivity and specificity for detecting reinfection compared to clinical and Ct value assessment when high-quality genomic data are available from multiple SARS-CoV-2 NAATs across illness episodes. Sequencing can also provide valuable information to inform both patient- and population-level decision making [5]. Viral genomic analysis can detect amino acid substitutions that may alter treatment guidelines, (e.g., suspension of bamlanivimab based on S:E484K prevalence [38, 39]). It can detect mutations evolving within immunocompromised individuals over time due to uncontrolled and potentially transmissible infection, as in subject P1 in our study [29], and enable consideration of SARS-CoV-2 retreatment. Population-level genomic surveillance may signal emergence of new variants with phenotypic differences in disease severity or transmissibility. In our study, three subjects with genomically-supported reinfection had notable substitutions in the spike or nucleocapsid proteins [21-24, 26]. Ongoing genomic surveillance of individuals with suspected SARS-CoV-2 reinfection is important to help understand whether mutations like these promote immune escape.

Despite the potential benefits of viral genomic analysis, this study highlighted ongoing obstacles to its use for routine differentiation of SARS-CoV-2 reinfection and persistent RNA detection. First, paired specimens from multiple infection episodes suitable for genomic analysis may be unavailable. Second, recovery of full SARS-CoV-2 genomes from specimens with lower viral RNA concentrations is difficult using conventional sequencing methods [19]. We recovered sufficient genomes in less than 25% of included subjects; most subjects with successful genome recovery had Ct values <30. Third, there is currently no automated method to perform genomic analysis of potential reinfections on a timeframe fast enough to guide clinical decision-making.

Overcoming these obstacles to mobilize viral genomic analysis for clinical care may be possible with substantial upfront investment. Access to sequencing and reduction in turnaround time could be achieved by embedding sequencing platforms and analyst personnel into clinical laboratories. Viral genomic yields could be improved by employing laboratory methods that reduce viral degradation (e.g., minimizing dwell time and freeze-thaw cycles) or deploying shorter-amplicon sequencing [40]. Independent replicates from source material may be employed to provide additional data to enhance genome coverage and sequencing depth.

This analysis has several important limitations. First, while this is among the largest SARS-CoV-2 reinfection cohort studies to combine detailed clinical and laboratory data with viral genomic evaluation, genomic analysis distinguished potential reinfection among only a modest number of subjects. Second, our cohort represents a convenience sample of individuals undergoing repeat SARS-CoV-2 NAAT as part of routine care. As such, we cannot provide a re-infection rate; however, our results demonstrate that reinfection can occur relatively soon after initial infection. Finally, this analysis was conducted before the emergence of SARS-CoV-2 variants of concern and deployment of COVID-19 vaccines and therapies. While these factors may influence reinfection risk and associated characteristics, this study provides a framework for using viral genomic analysis alongside clinical and Ct value assessment that could be adapted as the COVID-19 pandemic evolves.

**Conclusion**

While clinical and Ct value assessments were highly concordant with viral genomic analysis in identifying persistent RNA detection, they failed to identify a third of genomically-supported SARS-CoV-2 reinfections. Scaling-up viral genomic analysis for real-time clinical use would likely improve detection of SARS-CoV-2 reinfections, allow for treatment among the eligible, and provide much needed insights regarding evolution of variants of concern.

### NOTES

**ACKNOWLEDGEMENTS**

The authors thank Lauren West, MPH, for her assistance extracting data from the electronic health record and Andrea S. Foulkes, ScD for her contributions to the MGH COVID-19 Registry.

**FUNDING**

This work was supported by the Centers for Disease Control and Prevention [grant numbers U01CK000490 and U01CK000633 (E.T.R., E.P.H., S.E.T., R.C.C., and R.C.L.) and Broad Agency Announcement 75D30120C09605 to B.L.M] (also reported by J.E.L. and C.T-T.); National Institute of Allergy and Infectious Diseases [grant numbers U19AI110818 and U01AI151812 to P.C.S.; R01AI042006-24S1 to E.P.H. and I.V.B.]; the National Science Foundation Graduate Research Fellowship Program [grant number 1745303 to C.T.-T.]; National Cancer Institute [T32 CA 9216-41 to M.N.A.]; MGH DOM Physician-in-Chief, Dr. Katrina Armstrong's, COVID-19 Clinical Trial Fund and the MGH COVID Corps Program

[K.E.]; the Roger I. and Ruth B. MacFarlane Foundation [E.S.S.]; and the Howard Hughes Medical Institute [P.C.S.]. Any opinions, findings, and conclusions or recommendations expressed in this material are those of the author(s) and do not necessarily reflect the views of the National Science Foundation or the Centers for Disease Control and Prevention. The content is solely the responsibility of the authors and does not necessarily represent the official views of the National Institute of General Medical Sciences, the National Institutes of Health, or the Centers for Disease Control and Prevention. Funders had no role in the study's design, conduct, or reporting. C.T.-T. also reports COVID-19 baseline genomic surveillance contract to the Clinical Research Sequencing Platform of the Broad Institute of MIT and Harvard (grant no. 75D30121C10501) from Centers for Disease Control and Prevention. EPH also reports support for this manuscript paid to institution from CDC (GTEN).

### CONFLICTS OF INTEREST

MNA is an equity holder and consultant to Day Zero Diagnostics (company does not work on SARS-CoV-2). PCS is a co-founder of, shareholder in, and scientific advisor to Sherlock Biosciences, Inc (2019-present); she is also a Board member of and shareholder in Danaher Corporation (2019-present), and Co-Founder and Consultant to Delve Bio (2022-present). PCS has filed IP related to genome sequencing and analysis. PCS also reports that Sherlock Biosciences has licensed technology from the author and her lab on CRISPR-based diagnostics and has received consulting fees from the same; stock or stock options with Polaris Genomics (investor and future SAB member) and NextGen Jane (Former SAB member and investor). IVB reports an unrelated grant NIH R01AI042006-24S1. ESS reports unrelated grants or contracts from Centers for Disease Control and Prevention, Assistant Secretary for Preparedness and

Response, Massachusetts Institution of Technology/Quanta; payment or honoraria as a writer for Up To Date (2022 to current) and for a single lecture on COVID Infection Prevention to Vertex Pharmaceuticals, 2020; roles as President of Massachusetts Infectious Diseases Society, Vice-Chair of Public Policy and Government Affairs Committee, Society for Healthcare Epidemiology of America, and Member of Boston Biosafety Committee, Boston Public Health Commission. KAB reports NIH training grant 5T32 AI007387-32, unrelated to this work. CMD reports grants paid to institution (NIH/NICHD K08 HD101342; Harvard University CFAR; and MGH Executive Committee on Research), payments to institution from Centers for Disease Control and Prevention; and contracts to institution from International AIDS Vaccine Initiative and NIH/IMPAACT Network. RCL reports Grant from CDC: U01-CK000633; UpToDate: Royalties for Authorship; payment or honoraria from CDC Foundation: Editorial Services; a leadership or fiduciary role on the board of Greater Boston Physicians for Social Responsibility. JEL reports consulting fees 2019-2020 from Sherlock Biosciences; and honorarium from Virology Education. ER reports participation as PPD member of the Moderna Clinical Endpoint Adjudication Committee. SET reports royalties from UpToDate; travel support for attending Duke Clinical Research Institute meeting on preparing for the next pandemic; and reports that her husband is a partner and has equity in an economic consulting firm (Analysis Group Inc.) that provides consulting services for life science companies among many others. EPH reports grants or contracts unrelated to this work and paid to institution from NIH and MGH; royalties paid to author from UpToDate. All other authors report no conflicts of interest.

**Table 1. Cohort characteristics**

| | | Final categorization | | | | |
|---|---|---|---|---|---|---|
| **Characteristic** | **Total N=65** | **Genomically-supported reinfection N=6** | **Probable reinfection[Ψ] N=8** | **Probable persistent RNA detection N=18** | **Genomically-supported persistent RNA detection N=8** | **Inconclusive N=25** |
| Male sex, n (%) | 31 (48) | 5 (83) | 1 (13) | 7 (39) | 5 (63) | 13 (52) |
| Age, years, median (IQR) | 54 (31, 66) | 34 (28, 45) | 56 (51, 63) | 55 (33, 64) | 40 (24, 64) | 55 (31, 69) |
| Race/ethnicity | | | | | | |
| Asian, non-Hispanic | 3 (5) | 0 (0) | 0 (0) | 0 (0) | 1 (13) | 2 (8) |
| Black, non-Hispanic | 11 (17) | 2 (33) | 1 (13) | 2 (11) | 2 (25) | 4 (16) |
| Latinx/Hispanic | 30 (46) | 2 (33) | 4 (50) | 7 (39) | 4 (50) | 13 (52) |
| White, non-Hispanic | 18 (28) | 2 (33) | 3 (38) | 7 (39) | 1 (13) | 5 (20) |
| Other, non-Hispanic | 2 (3) | 0 (0) | 0 (0) | 2 (11) | 0 (0) | 0 (0) |
| Not reported | 1 (2) | 0 (0) | 0 (0) | 0 (0) | 0 (0) | 1 (4) |
| Healthcare worker with or without known exposure, n (%) | 10 (15) | 2 (33) | 1 (13) | 7 (39) | 0 (0) | 0 (0) |
| Immunocompromised, n (%)[¶] | 7 (11) | 0 (0) | 1 (13) | 2 (11) | 4 (50) | 0 (0) |
| Treatment received during initial episode, n (%) | | | | | | |
| Corticosteroids | 3 (5) | 0 (0) | 0 (0) | 1 (6) | 1 (13) | 1 (4) |
| Tocilizumab | 1 (2) | 0 (0) | 0 (0) | 1 (6) | 0 (0) | 0 (0) |
| Remdesivir | 3 (5) | 1 (17) | 0 (0) | 1 (6) | 0 (0) | 1 (4) |

| | | | | | | |
|---|---|---|---|---|---|---|
| Other[†] | 4 (6) | 0 (0) | 0 (0) | 1 (6) | 0 (0) | 3 (12) |
| COVID-19 treatment received during subsequent episode, n (%)[Φ] | 2 (3) | 0 (0) | 0 (0) | 0 (0) | 0 (0) | 2 (8) |
| Days between first and last positive test, median (IQR) | 65 (52, 89) | 117 (76, 237) | 87 (77, 111) | 55 (50, 65) | 60 (47, 70) | 61 (53, 94) |
| Ct value of first positive test, median (IQR) | 23.8 (17.5, 31.1) | 32.7 (25.0, 33.6) | 32.3 (31.2, 32.4) | 22.9 (15.8, 31.2) | 19.2 (16.7, 23.7) | 22.2 (17.2, 29.1) |
| Ct value of subsequent positive test, median (IQR) | 33.6 (31.8, 34.4) | 26.4 (22.7, 30.8) | 27.8 (21.0, 34.1) | 33.8 (33.5, 34.7) | 32.7 (29.2, 34.2) | 33.8 (32.4, 34.7) |
| Non-COVID respiratory pathogen identified, n (%)[‡] | 1 (2) | 0 (0) | 0 (0) | 1 (6) | 0 (0) | 0 (0) |
| Housing type at subsequent test, n (%) | | | | | | |
| Private home/apartment | 50 (77) | 4 (67) | 8 (100) | 14 (78) | 5 (63) | 19 (76) |
| Shelter or experiencing homelessness | 2 (3) | 1 (17) | 0 (0) | 0 (0) | 0 (0) | 1 (4) |
| Nursing home/assisted living/rehab | 8 (12) | 1 (17) | 0 (0) | 1 (6) | 1 (13) | 5 (20) |
| Not reported | 5 (8) | 0 (0) | 0 (0) | 3 (17) | 2 (25) | 0 (0) |
| Known household contact with COVID-19, n (%) | 1 (2) | 0 (0) | 1 (13) | 0 (0) | 0 (0) | 0 (0) |
| Admitted within 28 days of subsequent qualifying episode, n (%) | 20 (31) | 2 (33) | 1 (13) | 5 (28) | 2 (25) | 10 (40) |
| Required ICU care within 28 days, n (%) | 4 (6) | 0 (0) | 0 (0) | 1 (6) | 0 (0) | 3 (12) |
| Death within 28 days, n (%)[€] | 3 (5) | 1 (17) | 0 (0) | 1 (6) | 0 (0) | 1 (4) |

¶ Reasons for immunocompromised state included recent bone marrow transplant or use of any of the following: oral steroids, tacrolimus, mycophenolate, rituximab, azathioprine, or lenalinamide.
† Patients received one or more of: hydroxychloroquine or inhaled nitric oxide.
Φ Two subjects received corticosteroids for non-COVID-19 related indications. No other COVID-19 directed therapies were administered to subjects during their subsequent episode.
‡ One patient was found to have *Bordetella pertussis* on Biofire™ (Supplementary Methods) in the setting of persistent respiratory symptoms following an initial episode of COVID-19.
€ Cause of death included: ST-segment elevation myocardial infarction, decompensated cirrhosis, and recurrent stage IIIb non-small cell lung cancer with post-obstructive pneumonia. There were no deaths directly linked to COVID-19 infection.

[Ψ] One individual with probable reinfection reported travel to Florida in the month prior to initial diagnosis; no other individuals had reported travel history identified by chart review.

### FIGURE LEGENDS

**Figure 1. Identification of subjects meeting inclusion criteria.**

(A) Duration between the first and subsequent positive SARS-CoV-2 NAAT for all subjects with ≥1 SARS-CoV-2 NAAT collected at the Massachusetts General Hospital ≥ 1 day after an initial positive SARS-CoV-2 NAAT between March 14th and December 30th, 2020.

(B) Flow chart of subjects included in the study.

NAAT: nucleic acid amplification test; MGH: Massachusetts General Hospital; Ct: cycle threshold; LRT: lower respiratory tract; URT: upper respiratory tract

*Positive SARS-CoV-2 NAAT includes positive/detected and presumptive positive NAAT result interpretations

†Initial positive SARS-CoV-2 NAAT includes NAATs performed at an outside facility with results available in the MGH electronic medical record

**Figure 2. Comprehensive assessment of six subjects with genomically-supported SARS-CoV-2 reinfections in the context of Massachusetts (MA) COVID-19 cases.**

(A) Global phylogeny annotated with reinfection cases.

(B) Individual timelines of subjects with *genomically-supported reinfection*, MA COVID-19 case averages, and SARS-CoV-2 lineage prevalence in MA within the study duration. Individual timelines are annotated with nucleic acid amplification test results, cycle threshold values (when available), COVID-19 symptomatology, and SARS-CoV-2 PANGO lineages (when available).

Ct: cycle threshold; F: female; M: male; PMH: past medical history; MA: Massachusetts; NAAT: nucleic acid amplification test.

**Figure 3. Comprehensive assessment of eight subjects with genomically-supported persistent RNA detection in the context of Massachusetts (MA) COVID-19 cases.**

(A) Global phylogeny annotated with persistent RNA detection.

(B) Individual timelines of subjects with *genomically-supported persistent RNA detection* annotated with nucleic acid amplification test results, cycle threshold values (when available), COVID-19 symptomatology, and SARS-CoV-2 PANGO lineages (when available).

Ct: cycle threshold; M: male; DLBCL: diffuse large B-cell lymphoma; PMH: past medical history; DDKT: deceased donor kidney transplant; ESRD: end-stage renal disease; IS: immunosuppressed; F: female; ALL: acute lymphoblastic leukemia; BMT: bone marrow transplant; GVHD: graft versus host disease; CAD: coronary artery disease; CHF: congestive heart failure; ALS: amyotrophic lateral sclerosis; MA: Massachusetts; NAAT: nucleic acid amplification test.

**Figure 4. Comparison of cycle threshold (Ct) values between the first and subsequent (≥45 day) positive SARS-CoV-2 NAAT by final subject categorization.**

The lowest Ct value by final categorization for each subject is depicted. Of note, 12/65 (18%) subjects had a positive NAAT with a Ct value <35 at least 90 days after their initial positive test. Subject P4 had a day 90 NAAT with a Ct value of 32.81 but is represented in the 45-89 day group, because their lowest Ct value of 23.09 occurred on day 79. Subject I43 was excluded due to an unavailable Ct value for their day 237 positive NAAT. Ct: cycle threshold; NAAT: nucleic acid amplification test.

1

2 **Figure 1**

3 **165x170 mm ( x DPI)**

4

**Figure 2**
**261x252 mm ( x DPI)**

**Figure 3**
**232x251 mm ( x DPI)**

**Figure 4**
**156x101 mm ( x DPI)**

## Supplementary Material

ciac830_Supplementary_Data

