# Supplementary material for: Distinguishing Severe Acute Respiratory Syndrome Coronavirus 2 Persistence and Reinfection: A Retrospective Cohort Study
Source: Clin Infect Dis. 2022 Oct 21;76(5):850–60. doi: 10.1093/cid/ciac830 (PMC9619827; doi:10.1093/cid/ciac830)
Supplement: ciac830_Supplementary_Data [file ciac830_supplementary_data.docx]

**Distinguishing SARS-CoV-2 persistence and reinfection: A retrospective cohort study Supplemental Appendix**

**Supplementary Appendix Methods**

*Identification of subjects included in the cohort*

Using the Reporting Workbench tool, we queried the MGH Epic™ electronic health record to identify subjects with at least one SARS-CoV-2 NAAT test collected at a Massachusetts General Hospital (MGH) facility after any prior positive SARS-CoV-2 NAAT recorded in the Epic CareEverywhere system between March 14^th^ and December 30^th^, 2020 [1]. For the initial positive NAAT test, specimens were included irrespective of Ct value or specimen type. “Presumptive positive” NAAT results that occurred with the Cobas ® SARS-CoV-2 assays when the more sensitive pan-SARS target was positive, but the SARS-CoV-2-specific target was negative were considered to be positive for the purpose of inclusion criteria. The Epic CareEverywhere system provided access to COVID-19 testing history data from across 14 Boston-area healthcare systems [1]. As such, subjects could have had testing to diagnose initial infection at an outside institution, but they were still included in the study cohort if they had a positive test performed >45 days later at an MGH facility with residual specimen available in the MGH Microbiology Laboratory that could be used for viral genomic analysis.

*Reasons for repeat NAAT in the study period*

Common reasons for repeating NAAT after the initial positive test included assessment of new COVID-19 symptoms, admission or pre-procedural screening of asymptomatic individuals ≥ 90 days after initial COVID-19 diagnosis, and “test of cure” for resolution of transmission-based precautions or return to work. Of note, test-based resolution for purposes of ending isolation was largely replaced by time-based recommendations in summer of 2020, with test-based clearance applied in limited circumstances, based on CDC guidance.

*Cycle threshold value determination and standardization across platforms*

Cycle threshold values were obtained from clinical samples tested for routine clinical care and were generated by multiple test platforms deployed in the Clinical Microbiology Laboratory: cobas® SARS-CoV-2 test (Roche), Xpert® Xpress SARS-CoV-2 (Cepheid), Xpert Xpress SARS-CoV-2/Flu/RSV (Cepheid), and cobas® SARS-CoV-2 & Influenza A/B Test. In addition, for twenty-three positive samples without corresponding Ct values, available residual specimens were reanalyzed with the Taqpath^TM^ COVID-19 Combo Kit (Thermofisher) to obtain Ct values. To enable direct comparison of cycle thresholds between platforms, a set of specimens positive for SARS-CoV-2 were tested with the cobas® SARS-CoV-2 test in addition to each of the other test platforms. Linear regression analysis confirmed that Ct values generated using the Thermofisher instrument were significantly lower than those from the other RT-PCR platforms used in this study, which were not significantly different from each other. To correct for this variation between platforms, Ct values from the Thermofisher instrument were increased by 5.735, the y-intercept value. Since Ct values are the measurement most readily available to clinicians for real-time decision making, we present Ct values in lieu of viral titers. Additionally, the correlation between Ct values and RNA copies/ml for the Xpert Xpress and cobas platforms have been reported [2].

*Comparison of our inclusion criteria with CDC protocols*

In this analysis, any individual with an adjusted Ct value <35 from any NAAT target from a sample collected >45 days after an initial positive SARS-CoV-2 NAAT was considered to warrant further investigation for SARS-CoV-2 reinfection. These inclusion criteria were adapted from the CDC’s protocol for the investigation of SARS-CoV-2 reinfection (Supplemental Table 1) [3]. However, to increase sensitivity for detection of SARS-CoV-2 reinfection, our criteria for inclusion in the cohort deviated from CDC investigative criteria in three ways. First, for the subsequent positive NAATs, we used a Ct value cut-off of <35 for inclusion instead of <33 as recommended by the CDC. Second, COVID-19 symptoms were not required in individuals with detectable SARS-CoV-2 RNA 45-89 days after initial SARS-CoV-2 infection given the potential for asymptomatic reinfection. Third, we did not require that included participants have laboratory specimens available from the first episode of illness. For individuals whose specimens from the initial infection episode were not available, we compared genomic findings from the specimens obtained during subsequent episodes to variants known to be circulating in New England at the time of the initial episode. In these individuals, we also performed additional chart review to confirm that there was no documentation of recent travel outside of New England.

In addition to CDC criteria deviations implemented to increase sensitivity for detection of SARS-CoV-2 reinfection, we also required that the ≥45-day NAAT be performed on a nasopharyngeal (NP) or anterior nares (AN) specimen, because nucleic acid extraction procedures for genomic sequencing were not yet optimized on lower respiratory tract (LRT; i.e., tracheal aspirate, bronchoalveolar lavage, or expectorated sputum) specimens. Furthermore, we were concerned that severely ill individuals could demonstrate prolonged viral shedding in the lower respiratory tract (LRT) compared to the upper respiratory tract [4], which could bias our results, so individuals with only LRT tests from their subsequent testing episode were excluded.

*Assessment for non-SARS-CoV-2 respiratory viral pathogens*

We performed Biofire™ extended respiratory viral polymerase chain reaction (PCR) testing on an upper respiratory tract specimen obtained >45 days after the initial positive SARS-CoV-2 NAAT for all subjects meeting inclusion criteria who did not already have Biofire™ testing performed as part of their original clinical evaluation if available specimen was available. Since these Biofire results were not obtained as part of routine clinical care, the infectious disease physician reviewers who categorized included subjects by level of clinical suspicion for COVID-19 were blinded to these results at the time of their review.

*Viral genomic sequencing of clinical specimens*

We assessed viral titer and performed whole genome sequencing for all available samples from initial periods of positivity meeting inclusion criteria. We assembled genomes from all timepoints using the viral-ngs pipeline, aligning individual reads to the Wuhan-Hu-1 reference genome (GenBank accession NC_045512.2) [5, 6]. Nucleotide and amino substitutions relative to Wuhan-Hu-1were called for each subject using Nextclade [6]. Sequencing did not yield data for 27 samples from 18 patients, due to further sample degradation between aliquoting and library preparation (i.e., higher Ct values from additional freeze-thaw cycles), and an instrument failure affecting one sequencing run during the study period.

*Genomic classification of cases*

Phylogenies were assembled for each subject with sequences from its regional geographic context (MA, CT, RI, NH, VT, ME, and NY; published as of 2021-05-15) and close genetic proximity. Within the local tree for each patient, representative sequences were selected from both the initial infection and the later period of positivity based on completeness. Within each phylogeny, a node support metric (Shimodaira–Hasegawa–like approximate likelihood ratio test >80%) indicating phylogenetic separation of early and late genomes was used to identify reinfection. Genomes from reinfection cases satisfying these criteria were then also placed in a global tree of all SARS-CoV-2 sequences published on NCBI GenBank using UShER (Supplemental Table S6). Divergent placement (i.e., early and late genomes were placed on different local trees) corroborated reinfection in all instances identified using per-subject trees.

The series of viral genome assemblies for each subject was also evaluated for changes in PANGO lineage between early and late periods of positivity, or in the absence of data for the early period, presence of a lineage in the later period not yet in existence at the time of the earlier period of positivity, or at low frequency in the MA and the US at the time of sample collection. Apparent substitution rate per 30 days was calculated based on the number of nucleotide differences between representative genomes of the early and later periods of positivity. The representative genomes selected were those with the greatest number of called bases shared among others in the series, with preference given to earlier genomes of the early period, and later genomes of the late period, respectively, where the same bases were called in several genomes of the same time period. The genetic difference in number of substitutions was divided by the time elapsed between genomes being compared (in days), and multiplied by 30 to obtain an apparent mutation rate per 30 days.

Subject R2 had three nucleotide differences at 45 days (2 substitutions/30 days; 0.8x10^-3^ substitutions/site-year). While this substitution rate was near the population average of 1.1x10^-3^ substitutions/site-year [7], the specific substitutions characterized a distinct lineage circulating during the later period, suggesting a separate phylogenetic descent when compared with sequences from local and global contexts.

*Definition of immunocompromised state*

For the clinical characterization of the cohort, we defined “immunocompromised” based on the following Mass General Brigham criteria:

1. Immunocompromised based on diagnosis. Patients with any of the following diagnoses will be considered immunocompromised for the purposes of this policy.
   1. HIV CD4 <200
   2. Active lymphoma or leukemia (including indolent CLL)
   3. Metastatic cancer
   4. Cytotoxic chemotherapy within the prior 3 months
   5. Radiation therapy within prior 3 months
   6. Congenital immunodeficiency
   7. Aplastic anemia
   8. Solid organ transplant recipients on immunosuppressive therapy
   9. Hematopoietic stem cell transplant recipients, unless >2 years post-transplant AND no longer on any immunosuppressive therapy
2. Immunocompromised based on medications. Patients with receipt of the following medications will be considered immunocompromised for the purposes of this policy.
   1. Glucocorticoid therapy: the equivalent of prednisone 20 mg/d or more, for 2 weeks or more, or if such therapy has been discontinued within the past month
   2. Alkylating agents (e.g., cyclophosphamide) within the past 3 months
   3. Antimetabolites: methotrexate >0.4 mg/kg/week, azathioprine >3 mg/kg/day, 6- MP >1.5 mg/kg/day) within the past 3 months
   4. Cyclosporine, tacrolimus, sirolimus, everolimus, mycophenolate mofetil (usually given in the context of organ transplant, but sometimes used for other indications) within the past 3 months
   5. Biologic immunosuppressants and immunomodulators within the past 3 months (6 months for lymphocyte-depleting agents)

**Appendix Figure 1. Adjustment of cycle threshold values by test platform.** Comparison of cycle threshold values obtained from clinical samples tested with the Cobas SARS-CoV-2 test and one of the following assays: (A) Xpert® Xpress SARS-CoV-2 (Cepheid), (B) TaqPath COVID-19 Combo Kit, (C) Xpert Xpress SARS-CoV-2/Flu/RSV (Cepheid), and (D) cobas® SARS-CoV-2 & Influenza A/B Test. If the assay has multiple targets, the lowest Ct value for each sample was used in the analysis. The identity lines (y=x) and simple linear regression lines are shown for each graph.


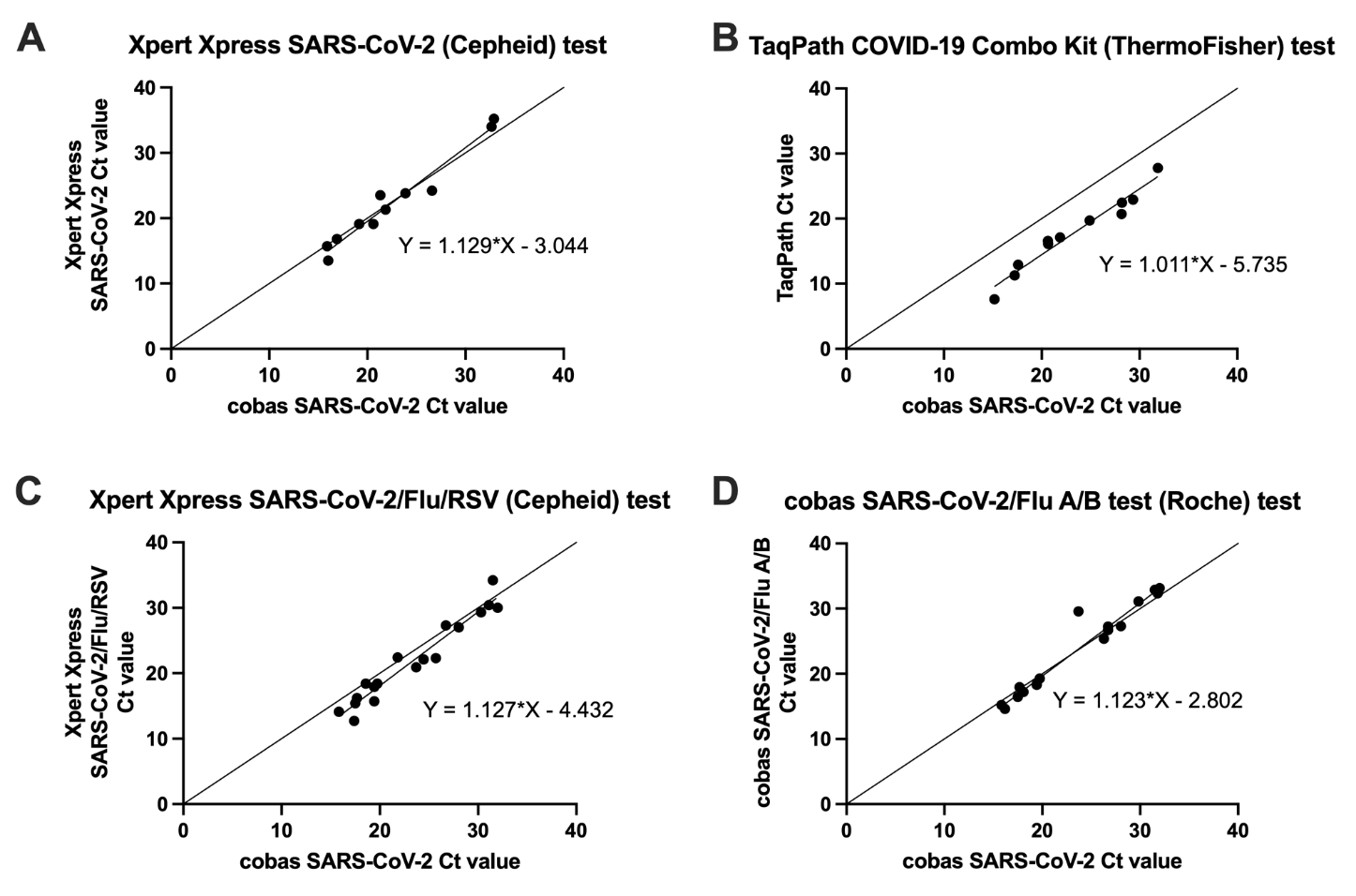


**Appendix Figure 2. Relationship between Ct value and unambiguous assembly length for all samples in the study for which genomic analysis was attempted.** Across diagnostic platforms, samples with a Ct value above 30 were significantly less likely to have complete genome coverage due to a low viral load (Ct < 30: median assembly length = 29,420 base pairs vs. Ct >30: median assembly length = 14,454 base pairs; p<0.0001 by Kruskal-Wallis test). Other studies have reported low overall genomic coverage with sequencing of samples that have a Ct >30 [8, 9]. However, sequencing of these samples was attempted in our study to provide the best chance at recovering viral sequence data for each sampled time point, some of which yielded specimens with high Ct values.


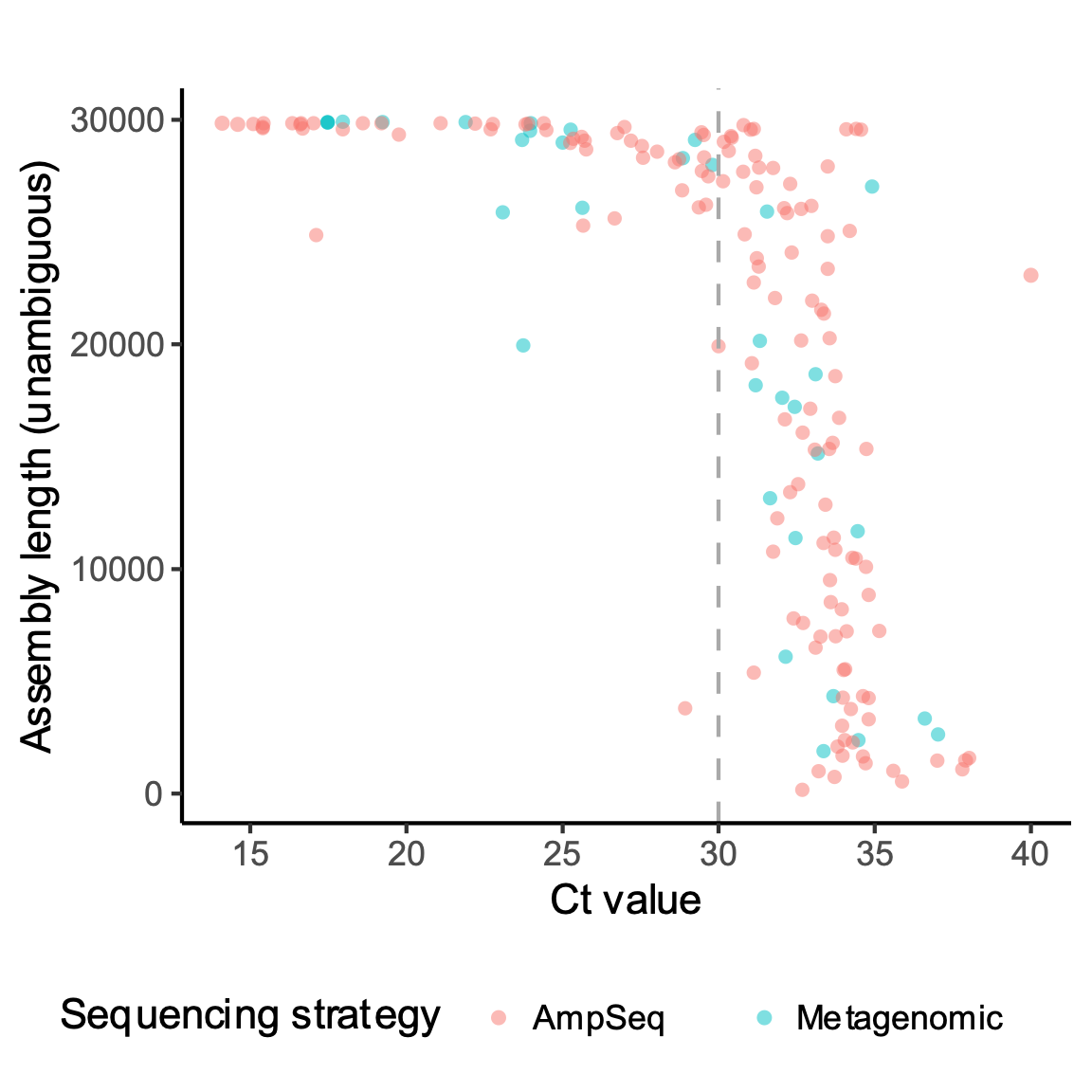


**Appendix Figure 3. Comparing the Ct value of the first positive NAAT and the subsequent NAAT with the lowest Ct value collected 45-89 days and >90 days after the first positive NAAT.** Symbols are colored based on clinical suspicion for reinfection. Subject I43 was excluded due to an unavailable Ct value for their day 237 positive NAAT.


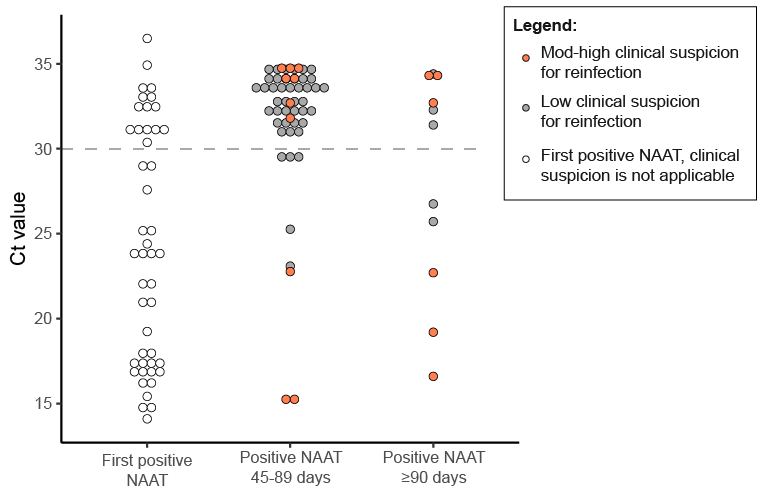


**Appendix Figure 4. Per-subject viral sequence alignments showing variation relative to the ancestral reference.** The top track represents the complete SARS-CoV-2 viral genome. The bases shown in the gray track below the genome indicate the ancestral allele present at each polymorphic position. Lower tracks correspond to viral sequences produced from each time point. Positions with conserved bases across all time points are omitted from the plot for legibility. Positions in timepoint tracks without colored boxes for bases maintain the ancestral allele. Substitutions are indicated with base-specific colors. Positions with “N” represent incomplete sequence coverage or ambiguous base calls.


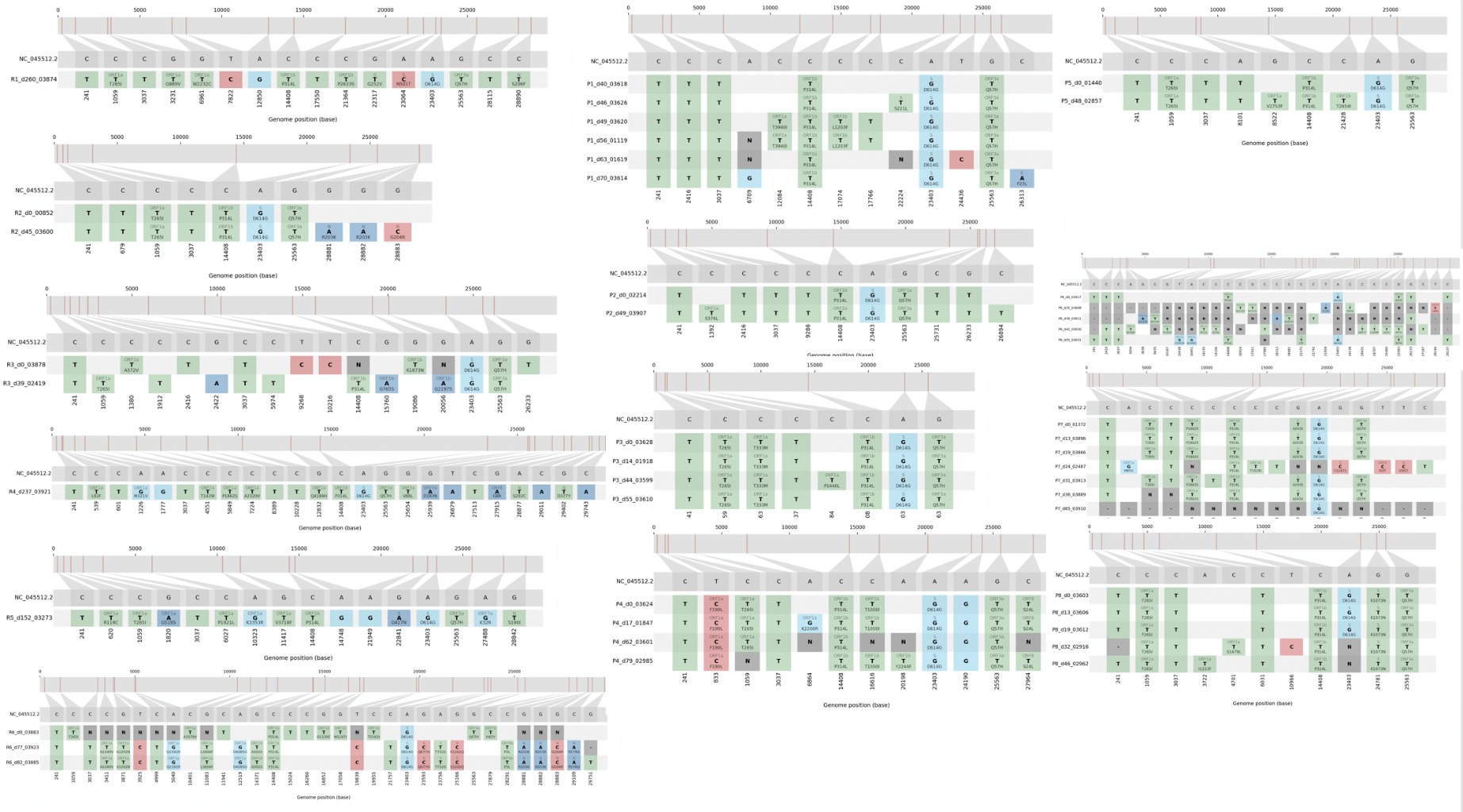


**Appendix Figure 5. Ct trajectories for subjects with *genomically-supported reinfection* (left) and *genomically-supported persistent RNA detection* (right).** Green circles indicate positive NAAT where a Ct value was available and vertical green lines indicate positive NAAT results for which Ct values were not available. Red circles represent negative NAAT results, which were assigned a Ct value of 45 for plotting purposes. Median Ct values from potential reinfection specimens were 26.4 (IQR: 22.7, 30.8) for subjects with *genomically-supported reinfection* and 32.7 (29.2, 34.2) for subjects with *genomically-supported persistent RNA detection* (p-value= 0.053).


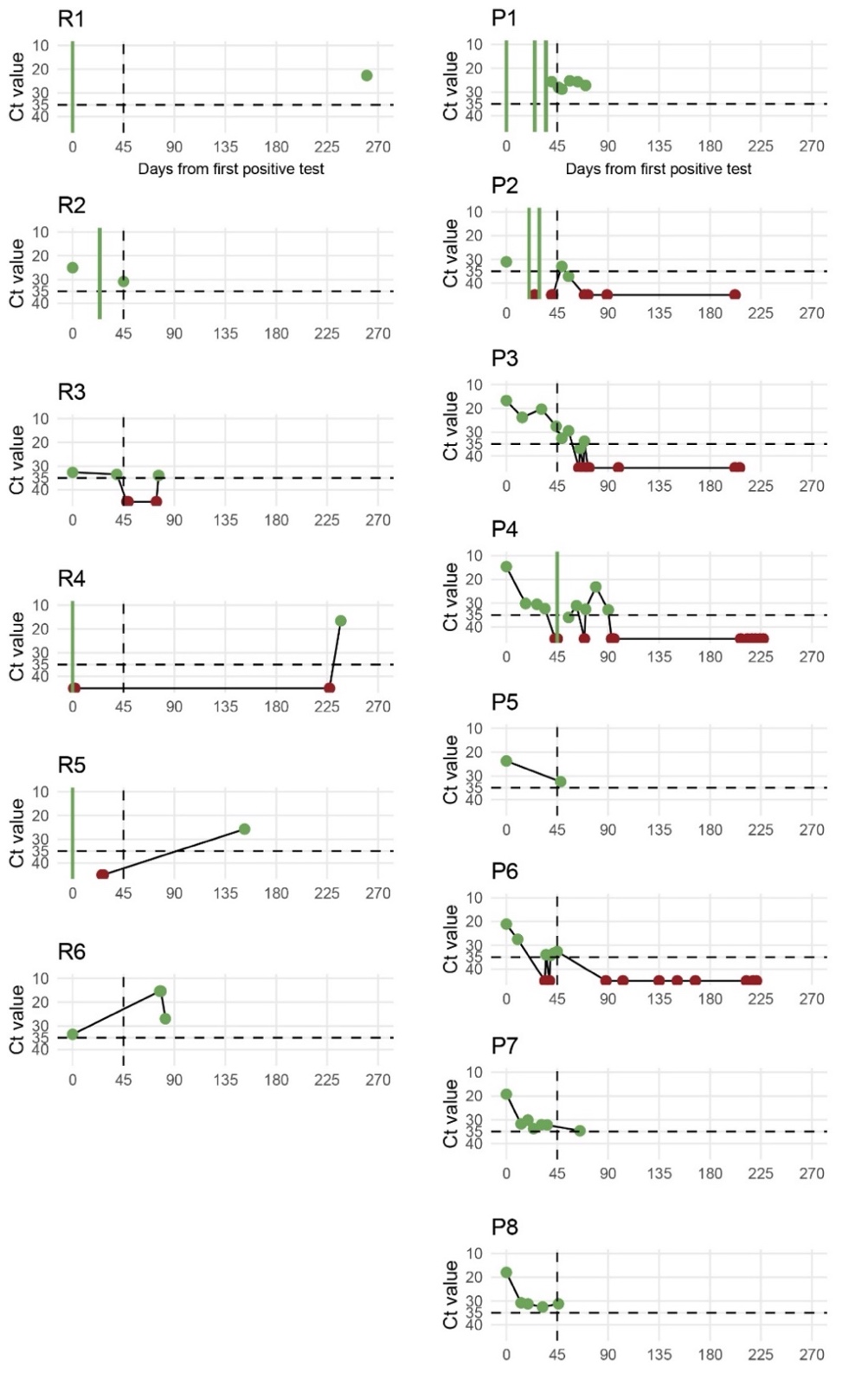


**Appendix Figure 6. Ct trajectories for the subjects with *probable reinfection*.** Green circles indicate positive NAAT where a Ct value was available and vertical green lines indicate positive NAAT results for which Ct values were not available. Red circles represent negative NAAT results, which were assigned a Ct value of 45 for plotting purposes.


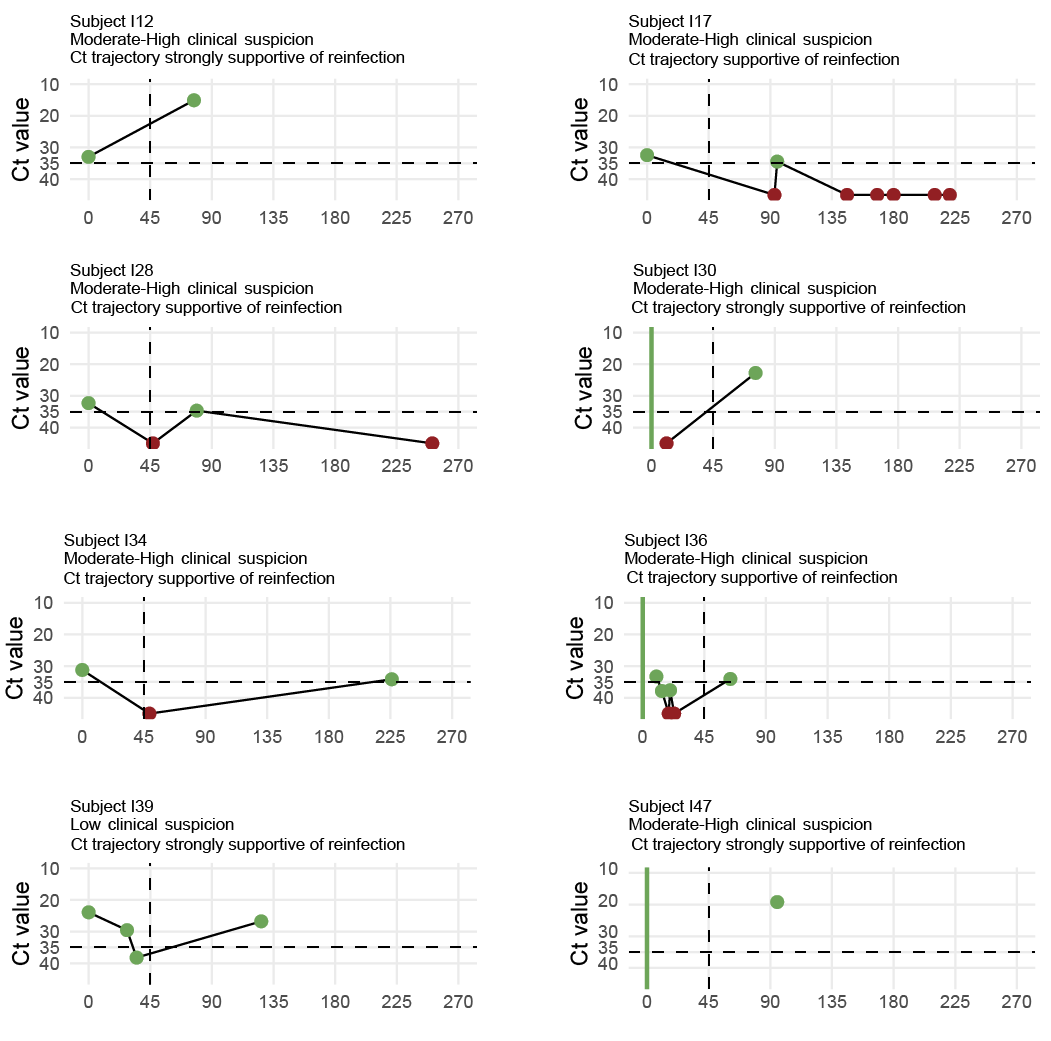


**Appendix Figure 7. Ct trajectories for the subjects with *probable persistent RNA detection* (unlikely reinfection).** Green circles indicate positive NAAT where a Ct value was available and vertical green lines indicate positive NAAT results for which Ct values were not available. Red circles represent negative NAAT results, which were assigned a Ct value of 45 for plotting purposes.


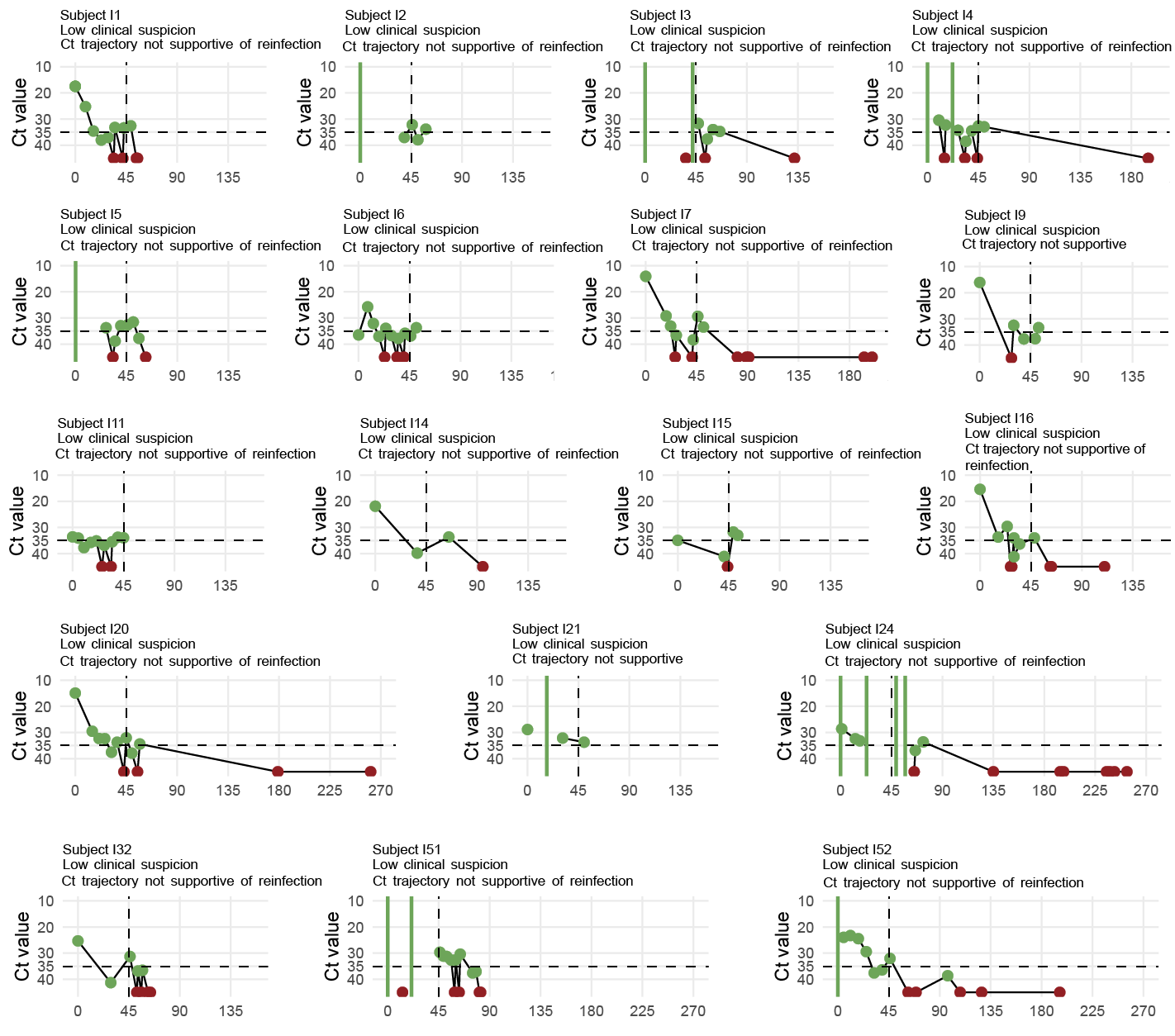


**Appendix Figure 8. Ct trajectories for the subjects with an *inconclusive* classification.** Green circles indicate positive NAAT where a Ct value was available and vertical green lines indicate positive NAAT results for which Ct values were not available. Red circles represent negative NAAT results, which were assigned a Ct value of 45 for plotting purposes.

**
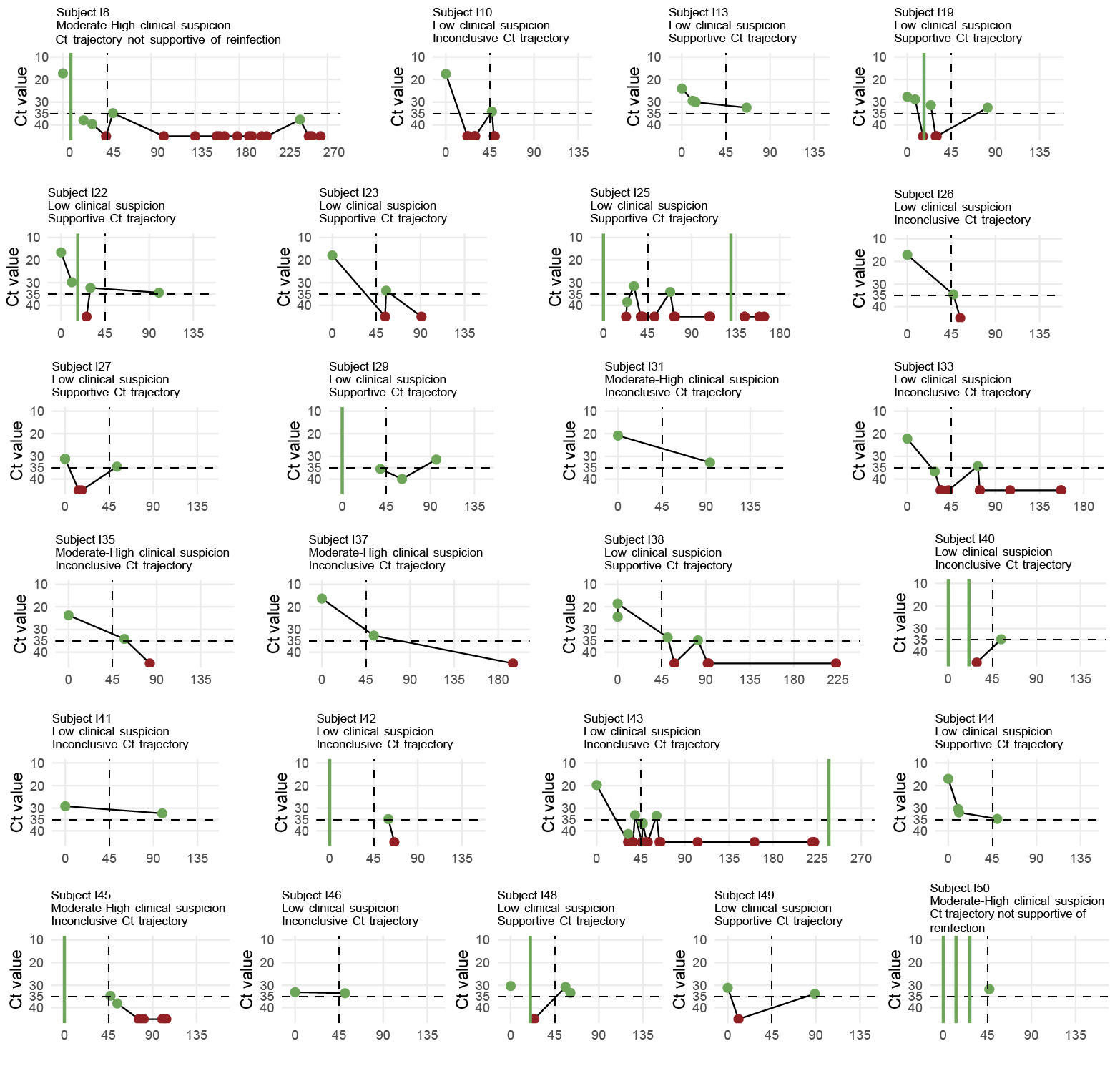
**

**Appendix Table 1. Clinical and laboratory investigative criteria for the identification of individuals with potential SARS-CoV-2 reinfection.**

| **CDC Criteria [3]** | **Study Criteria** |
| --- | --- |
| All persons with detectable SARS-CoV-2 RNA^*^ ≥ 90 days after initial SARS-CoV-2 infection  *or* | All persons with detectable SARS-CoV-2 RNA^†^ ≥ 45 days after initial SARS-CoV-2 positive test |
| Symptomatic persons with detectable SARS-CoV-2 RNA^*^ 45-89 days after initial SARS-CoV-2 infection PLUS at least one of the following:   - No alternative explanation for symptoms - Known close contact with a confirmed SARS-CoV-2-infected individual |  |
| *and exclude*  Persons for whom the laboratory specimen from either the first or second illness episode is unavailable | Including persons for whom laboratory specimens from either the first or second illness episode is unavailable, as genomic comparisons could be made against circulating strains in the community over time |

^*^Includes specimens with SARS-CoV-2 NAAT Ct values <33 or if values unavailable

^†^Includes specimens with SARS-CoV-2 NAAT Ct values <35

NAAT=nucleic acid test; Ct=cycle threshold

**Appendix Table 2. Cycle threshold value criteria for the classification of study subjects^*^**

| **Strong support for reinfection**   - Ct value <30 at least 45 days after initial positive NAAT and at least 30 days after last recent positive NAAT |
| --- |
| **Support for reinfection**   - Ct value ≥30 at least 45 days after first positive NAAT and at least 30 days after last recent positive NAAT that is within one 10-fold difference (3.3) in adjusted Ct value from the last NAAT (including intervening negative NAATs), suggesting an increasing viral load |
| **Not supportive of reinfection**   - Persistent Ct values that are at least 45 days after the first positive NAAT but less than 30 days after the last recent positive NAAT. |
| **Inconclusive**   - No Ct values available - Ct values at least 45 days after the first positive NAAT that are ≥30 with no points for comparison or ≥45 day Ct values not available - Ct value ≥30 at least 45 days after first positive NAAT and at least 30 days after last recent positive NAAT that is more than one 10-fold difference (3.3) higher than the adjusted Ct from the last NAAT (including intervening negative NAATs) - Ct value ≥30 at least 45 days after first positive NAAT and at least 30 days after last recent positive NAAT with a subsequent negative NAAT within 48 hours - Only 2 positive results available more than 30 days apart with last Ct value ≥30 |

^*^ Cycle threshold value criteria to evaluate for SARS-CoV-2 reinfection were extrapolated from definitions established by Abu-Raddad et al. [10].

**Appendix Table 3. Categorization rubric by clinical suspicion, cycle threshold assessment, and viral genomic analysis**

| **Category properties (subject must satisfy at least one criterion in each data type category)** | **Final reinfection categorization** | | | | | | |
| --- | --- | --- | --- | --- | --- | --- | --- |
|  | ***Genomically-supported reinfection*** | ***Probable reinfection*** | | ***Probable persistent RNA detection* (unlikely reinfection)** | ***Genomically-supported persistent RNA detection***  **(no reinfection)** | ***Inconclusive*** | |
| Clinical suspicion for reinfection | Any clinical assessment |  |  |  | Any clinical assessment |  |  |
| Moderate-high |  | X |  |  |  | X |  |
| Low |  |  | X | X |  |  | X |
| Cycle threshold value assessment | Any Ct value assessment |  |  |  | Any Ct value assessment |  |  |
| Strongly supportive of reinfection |  | X | X |  |  |  |  |
| Supportive of reinfection |  | X |  |  |  |  | X |
| Not supportive of reinfection |  |  |  | X |  | X |  |
| Inconclusive |  |  |  |  |  | X | X |
| Viral genomic analysis |  |  |  |  |  |  |  |
| Supportive of reinfection | X |  |  |  |  |  |  |
| Not supportive of reinfection |  |  |  |  | X |  |  |
| Inconclusive |  | X | X | X |  | X | X |

**Appendix Table 4. Substitutions observed in subjects with *genomically-supported reinfection* or *genomically-supported persistent RNA detection*.** We present all mutations observed in later genomes of subjects with *genomically-supported reinfection* and all substitutions observed in subjects with *genomically-supported persistent RNA detection* that emerged after the initial time point (i.e., *de novo* substitutions), relative to the ancestral reference genome.

| **Substitution** | **# Subjects with *genomically-supported reinfection* with specified mutation** | **# Subjects with *genomically-supported persistent RNA detection* with specified *de novo* mutation** |
| --- | --- | --- |
| ORF1b:P314L | 6 | 0 |
| S:D614G | 6 | 0 |
| ORF3a:Q57H | 5 | 0 |
| ORF1a:T265I | 4 | 0 |
| N:G204R | 2 | 0 |
| N:R203K | 2 | 0 |
| N:D377Y | 1 | 0 |
| N:P279Q | 1 | 0 |
| N:S190I | 1 | 0 |
| N:S202C | 1 | 0 |
| N:S206F | 1 | 0 |
| ORF1a:A1049V | 1 | 0 |
| ORF1a:A2320V | 1 | 0 |
| ORF1a:D4085G | 1 | 0 |
| ORF1a:G519S | 1 | 0 |
| ORF1a:G989V | 1 | 0 |
| ORF1a:K1202N | 1 | 0 |
| ORF1a:K3353R | 1 | 0 |
| ORF1a:L3606F | 1 | 0 |
| ORF1a:L92F | 1 | 0 |
| ORF1a:M321V | 1 | 0 |
| ORF1a:P1862S | 1 | 0 |
| ORF1a:P1921L | 1 | 0 |
| ORF1a:Q1592R | 1 | 0 |
| ORF1a:Q4189H | 1 | 0 |
| ORF1a:R119C | 1 | 0 |
| ORF1a:T1429I | 1 | 0 |
| ORF1a:V3718F | 1 | 0 |
| ORF1a:W2232C | 1 | 0 |
| ORF1b:A302S | 1 | 0 |
| ORF1b:G2197S | 1 | 0 |
| ORF1b:G765S | 1 | 0 |
| ORF1b:P2633S | 1 | 0 |
| ORF3a:D183N | 1 | 0 |
| ORF3a:V88L | 1 | 0 |
| ORF7a:K32R | 1 | 0 |
| ORF8:G8R | 1 | 0 |
| ORF9b:P3L | 1 | 0 |
| S:D427N | 1 | 0 |
| S:E1202Q | 1 | 0 |
| S:G252V | 1 | 0 |
| S:N501T | 1 | 0 |
| S:Q677H | 1 | 0 |
| S:T732S | 1 | 0 |
| E:F23L | 0 | 1 |
| N:I292T | 0 | 1 |
| ORF1a:C3301F | 0 | 1 |
| ORF1a:C3391W | 0 | 1 |
| ORF1a:G1125S | 0 | 1 |
| ORF1a:I1153F | 0 | 1 |
| ORF1a:K2200R | 0 | 1 |
| ORF1a:M85V | 0 | 1 |
| ORF1a:N1080I | 0 | 1 |
| ORF1a:N3396D | 0 | 1 |
| ORF1a:P1640L | 0 | 1 |
| ORF1a:Q2724* | 0 | 1 |
| ORF1a:S376L | 0 | 1 |
| ORF1a:T3940I | 0 | 1 |
| ORF1a:V2753F | 0 | 1 |
| ORF1b:H1153Y | 0 | 1 |
| ORF1b:L1203F | 0 | 1 |
| ORF1b:P223L | 0 | 1 |
| ORF1b:R1315L | 0 | 1 |
| ORF1b:S230L | 0 | 1 |
| ORF1b:T1529I | 0 | 1 |
| ORF1b:Y2244F | 0 | 1 |
| ORF3a:A23V | 0 | 1 |
| ORF6:I33T | 0 | 1 |
| ORF8:L84S | 0 | 1 |
| S:F133I | 0 | 1 |
| S:L1049F | 0 | 1 |
| S:L5F | 0 | 1 |
| S:Q957* | 0 | 1 |
| S:S221L | 0 | 1 |
| S:T859I | 0 | 1 |

**Appendix Table 5. Categorization of subjects by clinical suspicion, cycle threshold value assessment, and viral genomic analysis**

| **Classification of subjects in study by data type** | **Total**  **N=65** | **Final reinfection categorization** | | | | |
| --- | --- | --- | --- | --- | --- | --- |
|  |  | ***Genomically-supported reinfection* N=6** | ***Probable reinfection* N=8** | ***Probable persistent RNA detection* (unlikely reinfection)**  **N=18** | ***Genomically-supported persistent RNA detection***  **(no reinfection)**  **N=8** | ***Inconclusive* N=25** |
| Clinical suspicion for reinfection |  |  |  |  |  |  |
| Moderate-high | 17 | 3 | 7 | 0 | 0 | 7 |
| Low | 48 | 3 | 1 | 18 | 8 | 18 |
| Cycle threshold value assessment |  |  |  |  |  |  |
| Strongly supportive of reinfection | 8 | 4 | 4 | 0 | 0 | 0 |
| Supportive of reinfection | 17 | 1 | 4 | 0 | 0 | 12 |
| Not supportive of reinfection | 28 | 1 | 0 | 18 | 7 | 2 |
| Inconclusive | 12 | 0 | 0 | 0 | 1 | 11 |
| Viral genomic analysis |  |  |  |  |  |  |
| Supportive of reinfection | 6 | 6 | 0 | 0 | 0 | 0 |
| Not supportive of reinfection | 8 | 0 | 0 | 0 | 8 | 0 |
| Inconclusive | 51 | 0 | 8 | 18 | 0 | 25 |

**Appendix Table 6. NCBI BioSample accessions**

| **Patient identifier with day of collection and specimen number** | **NCBI BioSample accession** |
| --- | --- |
| R1_d260_03874 | SAMN17906045 |
| R2_d0_00852 | SAMN15752074 |
| R2_d45_03600 | SAMN17824041 |
| R3_d0_03878 | SAMN17906049 |
| R3_d39_02419 | SAMN15753712 |
| R4_d237_03921 | SAMN17906092 |
| R5_d152_03273 | SAMN17211223 |
| R6_d0_03883 | SAMN17906054 |
| R6_d77_03923 | SAMN17906094 |
| R6_d78_04138 | SAMN18858631 |
| R6_d82_03885 | SAMN17906056 |
| P1_d40_03618 | SAMN17824059 |
| P1_d46_03626 | SAMN17824067 |
| P1_d49_03620 | SAMN17824061 |
| P1_d56_01119 | SAMN15752341 |
| P1_d63_01619 | SAMN15752919 |
| P1_d70_03614 | SAMN17824055 |
| P2_d0_02214 | SAMN15753514 |
| P2_d49_03907 | SAMN17906078 |
| P3_d0_03628 | SAMN17824069 |
| P3_d14_01918 | SAMN15753218 |
| P3_d44_03599 | SAMN17824040 |
| P3_d55_03610 | SAMN17824051 |
| P4_d0_03624 | SAMN17824065 |
| P4_d17_01847 | SAMN15753147 |
| P4_d62_03601 | SAMN17824042 |
| P4_d79_02985 | SAMN17210821 |
| P5_d0_01440 | SAMN15752740 |
| P5_d48_02857 | SAMN17210693 |
| P6_d0_03917 | SAMN17906088 |
| P6_d35_03608 | SAMN17824049 |
| P6_d39_03611 | SAMN17824052 |
| P6_d42_03630 | SAMN17824071 |
| P6_d45_03631 | SAMN17824072 |
| P7_d0_01372 | SAMN15752672 |
| P7_d13_03896 | SAMN17906067 |
| P7_d19_03866 | SAMN17906037 |
| P7_d24_02487 | SAMN15753780 |
| P7_d31_03913 | SAMN17906084 |
| P7_d36_03889 | SAMN17906060 |
| P7_d65_03910 | SAMN17906081 |
| P8_d0_03603 | SAMN17824044 |
| P8_d13_03606 | SAMN17824047 |
| P8_d19_03612 | SAMN17824053 |
| P8_d32_02916 | SAMN17210752 |
| P8_d46_02962 | SAMN17210798 |
| I1_d0_00147 | SAMN14938727 |
| I1_d0_00090 | SAMN14938698 |
| I1_d9_03615 | SAMN17824056 |
| I1_d16_02175 | SAMN15753475 |
| I1_d23_03622 | SAMN17824063 |
| I1_d29_03632 | SAMN17824073 |
| I1_d35_01678 | SAMN15752978 |
| I1_d43_01496 | SAMN15752796 |
| I2_d39_01435 | SAMN15752735 |
| I2_d46_01485 | SAMN15752785 |
| I2_d51_02035 | SAMN15753335 |
| I2_d58_03879 | SAMN17906050 |
| I3_d0_00536 | SAMN14939011 |
| I3_d47_01330 | SAMN15752630 |
| I3_d60_03905 | SAMN17906076 |
| I3_d66_03871 | SAMN17906042 |
| I4_d10_02191 | SAMN15753491 |
| I4_d27_03619 | SAMN17824060 |
| I4_d39_01422 | SAMN15752722 |
| I4_d50_03616 | SAMN17824057 |
| I5_d27_03906 | SAMN17906077 |
| I5_d51_01628 | SAMN15752928 |
| I5_d56_02104 | SAMN15753404 |
| I6_d8_02228 | SAMN15753528 |
| I6_d24_03897 | SAMN17906068 |
| I6_d41_02045 | SAMN15753345 |
| I6_d51_03869 | SAMN17906040 |
| I7_d0_03928 | SAMN17906099 |
| I7_d18_01548 | SAMN15752848 |
| I7_d22_01128 | SAMN15752350 |
| I7_d27_01474 | SAMN15752774 |
| I7_d46_03865 | SAMN17906036 |
| I7_d51_02509 | SAMN15753802 |
| I8_d51_03908 | SAMN17906079 |
| I9_d30_01581 | SAMN15752881 |
| I9_d52_03886 | SAMN17906057 |
| I10_d0_00149 | SAMN14938729 |
| I10_d47_03920 | SAMN17906091 |
| I11_d5_03877 | SAMN17906048 |
| I11_d21_01677 | SAMN15752977 |
| I11_d45_03867 | SAMN17906038 |
| I12_d77_03607 | SAMN17824048 |
| I13_d0_01011 | SAMN15752233 |
| I13_d14_03919 | SAMN17906090 |
| I13_d66_03914 | SAMN17906085 |
| I14_d0_00602 | SAMN14939077 |
| I14_d65_02537 | SAMN15753830 |
| I15_d0_00078 | SAMN14938686 |
| I15_d49_01961 | SAMN15753261 |
| I15_d53_03894 | SAMN17906065 |
| I16_d0_03892 | SAMN17906063 |
| I16_d16_01234 | SAMN15752534 |
| I16_d24_03931 | SAMN17906102 |
| I16_d48_03903 | SAMN17906074 |
| I17_d0_00790 | SAMN15752012 |
| I17_d95_03000 | SAMN17210842 |
| I19_d0_03627 | SAMN17824068 |
| I19_d8_03623 | SAMN17824064 |
| I19_d24_01489 | SAMN15752789 |
| I20_d15_02299 | SAMN15753599 |
| I20_d21_03899 | SAMN17906070 |
| I20_d26_03888 | SAMN17906059 |
| I20_d37_01676 | SAMN15752976 |
| I20_d45_01466 | SAMN15752766 |
| I20_d57_03902 | SAMN17906073 |
| I21_d0_00813 | SAMN15752035 |
| I21_d31_02388 | SAMN15753688 |
| I21_d50_02410 | SAMN15753703 |
| I22_d0_03900 | SAMN17906071 |
| I22_d11_01323 | SAMN15752623 |
| I22_d30_03872 | SAMN17906043 |
| I22_d100_03924 | SAMN17906095 |
| I23_d0_00627 | SAMN14939102 |
| I23_d55_03868 | SAMN17906039 |
| I24_d1_03929 | SAMN17906100 |
| I24_d17_00965 | SAMN15752187 |
| I24_d73_03864 | SAMN17906035 |
| I25_d68_02435 | SAMN15753728 |
| I26_d0_02120 | SAMN15753420 |
| I26_d47_03863 | SAMN17906034 |
| I27_d0_02850 | SAMN17210686 |
| I27_d0_03613 | SAMN17824054 |
| I28_d79_02851 | SAMN17210687 |
| I29_d39_02576 | SAMN15753869 |
| I29_d61_03918 | SAMN17906089 |
| I29_d96_02866 | SAMN17210702 |
| I30_d76_03873 | SAMN17906044 |
| I31_d94_03634 | SAMN17824075 |
| I32_d0_04135 | SAMN18858628 |
| I32_d46_02557 | SAMN15753850 |
| I33_d0_03930 | SAMN17906101 |
| I33_d72_03925 | SAMN17906096 |
| I34_d0_02377 | SAMN15753677 |
| I35_d0_03193 | SAMN17211035 |
| I35_d57_03882 | SAMN17906053 |
| I36_d10_03893 | SAMN17906064 |
| I36_d64_03911 | SAMN17906082 |
| I37_d0_03629 | SAMN17824070 |
| I37_d53_02883 | SAMN17210719 |
| I38_d0_03887 | SAMN17906058 |
| I38_d0_03887 | SAMN17906058 |
| I38_d51_02413 | SAMN15753706 |
| I38_d82_03926 | SAMN17906097 |
| I39_d0_04137 | SAMN18858630 |
| I39_d28_04136 | SAMN18858629 |
| I39_d126_04134 | SAMN18858627 |
| I40_d54_03881 | SAMN17906052 |
| I42_d60_03927 | SAMN17906098 |
| I43_d0_02344 | SAMN15753644 |
| I43_d39_03904 | SAMN17906075 |
| I43_d61_03891 | SAMN17906062 |
| I44_d0_03876 | SAMN17906047 |
| I44_d10_03895 | SAMN17906066 |
| I44_d11_03358 | SAMN17211308 |
| I44_d50_03884 | SAMN17906055 |
| I46_d0_03909 | SAMN17906080 |
| I46_d51_03912 | SAMN17906083 |
| I47_d95_03933 | SAMN17906104 |
| I48_d56_03932 | SAMN17906103 |
| I48_d61_03870 | SAMN17906041 |
| I49_d0_03880 | SAMN17906051 |
| I49_d89_03875 | SAMN17906046 |
| I50_d47_03934 | SAMN17906105 |
| I51_d46_03602 | SAMN17824043 |
| I51_d49_02513 | SAMN15753806 |
| I51_d52_02469 | SAMN15753762 |
| I51_d56_03609 | SAMN17824050 |
| I51_d64_04233 | SAMN18515258 |
| I52_d5_01593 | SAMN15752893 |
| I52_d18_04139 | SAMN18858632 |
| I52_d25_04140 | SAMN18858633 |

**REFERENCES:**

1. Epic. Organizations on the Care Everywhere network. Available at: <https://www.epic.com/careeverywhere/>. Accessed December 3 2021.

2. Fajnzylber J, Regan J, Coxen K, et al. SARS-CoV-2 viral load is associated with increased disease severity and mortality. Nature Communications **2020**; 11:5493.

3. Centers for Disease Control and Prevention. Common investigation protocol for investigating suspected SARS-CoV-2 reinfection Available at: <https://www.cdc.gov/coronavirus/2019-ncov/php/reinfection.html>. Accessed October 27 2020.

4. Chen PZ, Bobrovitz N, Premji ZA, Koopmans M, Fisman DN, Gu FX. SARS-CoV-2 shedding dynamics across the respiratory tract, sex, and disease severity for adult and pediatric COVID-19. Elife **2021**; 10.

5. Genbank. Severe acute respiratory syndrome coronavirus 2 isolate Wuhan-Hu-1, complete genome. Available at: <https://www.ncbi.nlm.nih.gov/nuccore/NC_045512.2>. Accessed October 7 2021.

6. Aksamentov I, Roemer C, Hodcroft EB, Neher RA. Nextclade: clade assignment, mutation calling and quality control for viral genomes. J Open Source Softw **2021**; 6:1-5.

7. Duchene S, Featherstone L, Haritopoulou-Sinanidou M, Rambaut A, Lemey P, Baele G. Temporal signal and the phylodynamic threshold of SARS-CoV-2. Virus Evol **2020**; 6:veaa061.

8. Lemieux JE, Siddle KJ, Shaw BM, et al. Phylogenetic analysis of SARS-CoV-2 in Boston highlights the impact of superspreading events. Science **2021**; 371.

9. Tyson JR, James P, Stoddart D, et al. Improvements to the ARTIC multiplex PCR method for SARS-CoV-2 genome sequencing using nanopore. bioRxiv **2020**:2020.09.04.283077.

10. Abu-Raddad LJ, Chemaitelly H, Malek JA, et al. Assessment of the risk of severe acute respiratory syndrome coronavirus 2 (SARS-CoV-2) reinfection in an intense reexposure setting. Clin Infect Dis **2021**; 73:e1830-e40.
